# 

**Published:** 1843-02-18

**Authors:** 


**And Retrospect of the Medical Sciences.**

EDITED BY

MEREDITH CLYMER, M.D.

VOL. VI.] PHILADELPHIA, FEBRUARY 18, 1843. [No. 3.

### CONTENTS.

A Course of Clinical Lectures, delivered at the Hôtel-Dieu, Paris, for the Session 1842–'43. By A. F. Chomel. Lecture 2.
On hepatic colic, . . . . 25

*Jefferson Medical College.*
Clinic of Professor Mütter.
Case 2. Epulis, developed in the substance of the gum, and originating in neglected parulis, . . . . 27

*University of Pennsylvania.*
Clinic of Dr. W. Poyntell Johnston.
Case 1. Granular blepharitis following measles—application of solid sulphate of copper, . . . . . 30
Case 2. Long continued masturbation—granular condition of the mucous membrane of the prostatic portion of the urethra—nocturnal and diurnal pollutions—imbecility—hypochondriasis—application of the solid nitrate of silver, . . . . 30

*Bibliographical Notices.*
Lecture on the diseases of the urinary organs. By Sir Benjamin C. Brodie, Bart. F.R.S. Serj ant Surgeon to the Queen. From the third London edition, with alterations and additions. Philadelphia: 1843. 8vo. pp. 214, . . . . . . 33
The Diseases of Females: including those of Pregnancy and Childbed. By Fleetwood Churchill, M.D., etc. etc. Second American Edition. With notes. By Robert M. Huston, M. D., Professor of Materia Medica and Therapeutics in the Jefferson Medical College, etc. etc. Philadelphia: 1843. 8vo. pp. 575. . 34
A Practical Treatise on Venereal Disease; or, Critical and Experimental Researches on Innoculation, applied to the Study of these Affections; with a Therapeutic Summary and Special Formulary. By Ph. Ricord, M.D., Surgeon of the Venereal Hospital at Paris, etc. etc. Translated from the French. By Henry Pilkington Drummond, M. D. Philadelphia: 1843. 8vo. pp. 2[illegible]6. . 34
Dr. Hayward on the statistics of pulmonary consumption, . . . 34

*Retrospect of the Medical Sciences.*
Successful operation for strangulated hernia in an infant seven weeks old. By Mr. Cæsar Hawkins, . 35
Fracture of the clavicle without displacement, . . . . . . 35
The diseases of nightmen, . . 36
Morbid anatomy of the nervous system 36
Iridoplasma, . . . . . . 36

